# Effect of low-power diode laser on infected root canals

**DOI:** 10.1590/0103-6440202204999

**Published:** 2022-06-24

**Authors:** Denise Ramos Silveira Alves, Daniel de Almeida Decucio, Ana Helena Gonçalves de Alencar, Cyntia Rodrigues de Araújo Estrela, João Batista de Souza, Antônio Luiz Barbosa Pinheiro, Carlos Estrela

**Affiliations:** 1 Faculty of Dentistry, Federal University of Goiás, Goiânia, GO, Brazil; 2 School of Dentistry, UniEvangélica, Anápolis, GO, Brazil.; 3 School of Dentistry, Federal University of Bahia, Salvador, BA, Brazil

**Keywords:** Photodynamic therapy, Enterococcus faecalis, biofilm, methylene blue

## Abstract

This study evaluated the effect of photodynamic therapy (PDT) on infected root canals. Twenty-one human teeth were selected, and 18 were infected by *E. faecalis* for 60 days. The antimicrobial strategies tested were: G1. Root canal preparation (RCP) using Niquel-Titanium (NiTi) rotary instruments, 2.5% NaOCl, and final irrigation with 17% EDTA, followed by PDT with methylene blue photosensitizer and laser diode low power; G2. RCP using stainless steel files and the same irrigation and PDT protocols as G1; G3. Same RCP protocol as G1 without PDT; G4. Only irrigation with 2.5% NaOCl; G5. Same PDT protocol as G1 without RCP; G6. Negative control; G7. Positive control. Samples for microbiological tests were collected initially (S1), after RCP (S2), and after PDT (S3). Subsequently, the roots were sectioned and prepared for Scanning Electron Microscopy (SEM) analysis. Bacterial growth was analyzed according to the turbidity of the culture medium, followed by spectrophotometric optical density (nm). The effect of PDT on the dentinal structure was evaluated at magnifications 1,600X and 5,000X and described qualitatively. The Wilcoxon test was used for the comparisons from the same specimens, and the Mann-Whitney test was used to compare groups ((=5%). Bacteria were found in all experimental groups’ microbiological samples (S1, S2 and S3). The optical density of culture media was lower in S2 than in S1 of G1, 2, 3, and 4 (p> 0.05). After PDT (S3) in G1 and 2, there was an additional reduction in optical density of the culture medium, respectively (p>0.05). In Group 5, the analysis of culture media at S2 revealed an increase in optical density compared to S1(p>0.05). In SEM images of G1, 2, and 5, dentin with melting and recrystallization areas were evidenced. After preparation of the root canal with the rotary system or manually associated with 2.5% NaOCl, PDT was not able to completely eliminate *E. faecalis* present in the root canal.

## Resumo

Este estudo avaliou o efeito da terapia fotodinâmica (PDT) em canais radiculares infectados com *E. faecalis*. Vinte e um dentes humanos extraídos foram selecionados, e 18 foram infectados por *E. faecalis* por 60 dias. As estratégias antimicrobianas testadas foram: G1. Preparo do canal radicular (PCR) com instrumentos rotatórios de NiTi, NaOCl 2,5% e irrigação final com EDTA 17%, seguido de PDT com fotossensibilizador azul de metileno e laser diodo de baixa potência; G2. PCR usando limas de aço inoxidável e os mesmos protocolos de irrigação e PDT do G1; G3. Protocolo de PCR similar que G1 sem PDT; G4. Somente irrigação com NaOCl 2,5%; G5. Protocolo similar ao G1, sem PCR; G6. Controle negativo; G7. Controle positivo. Amostras para exames microbiológicos foram coletadas inicialmente (S1), após PCR (S2) e após PDT (S3). Na sequência, as raízes foram seccionadas e preparadas para análise em microscopia eletrônica de varredura (MEV). O crescimento bacteriano foi analisado de acordo com a turbidez do meio de cultura seguida pela densidade óptica espectrofotométrica (nm). O efeito da PDT na estrutura dentinária foi avaliado em aumentos de 1.600X e 5.000X, e descrito qualitativamente. O teste de Wilcoxon foi utilizado para as comparações dos mesmos espécimes e o teste de Mann-Whitney para as comparações entre os grupos ((=5%). Bactérias foram encontradas em todos os grupos experimentais, e em todas as coletas microbiológicas (S1, S2 e S3). A densidade óptica dos meios de cultura foi menor em S2 do que em S1 de G1, 2, 3 e 4 (p>0,05). Após a PDT (S3) em G1 e 2, houve redução adicional na densidade óptica do meio de cultura de 90,0% e 92,0%, respectivamente (p>0,05). No Grupo 5, a análise dos meios de cultura em S2 revelou um aumento de 3,2% na densidade óptica em comparação com S1(p>0,05). Nas imagens de MEV do G1, 2 e 5 foram evidenciadas dentina com áreas de fusão e recristalização. O PDT utilizado após preparo do canal radicular com sistema rotatório ou manual, associado ao NaOCl 2,5%, não foi capaz de eliminar completamente o *E. faecalis* em biofilme maduro presente no canal radicular.

## Introduction

The effective removal of bacterial biofilm from root canals, an important concern in Endodontics, is directly associated with endodontic success rates ^1^ ^,^ ^2^. Therefore, the most frequent antimicrobial strategy in endodontic therapy is cleaning and shaping, which combines the action of endodontic instruments with irrigants, particularly sodium hypochlorite (NaOCl) and irrigant agitation, which intensifies the antibacterial effect. However, the complex anatomy of the root canal system is a challenging factor for antibacterial strategies, and full sanitization is challenging to achieve, even when irrigants and intracanal medications are used ^2^.

In addition to instrumentation, complementary strategies have been suggested to increase disinfection of the root canal system as photodynamic therapy (PDT) ^3^ ^,^ ^4^ ^,^ ^5^ ^,^ ^6^ ^,^ ^7^ ^,^ ^8^ ^,^ ^9^ ^,^ ^10^. Despite a relative long-term use, PDT still attracts attention because of the increasing resistance to antibiotics worldwide. PDT is based on nontoxic dyes, known as photosensitizers (PS) that interact with the target cells and undergo excitation in the presence of visible light of an adequate wavelength. As a consequence of the PS-light interaction, reactive oxygen species (ROS), free radicals, or singlet oxygen (_1_O^2^) may be generated. They all affect different bacterial structures and kill them by damaging their cytoplasm membranes or DNA ^3^. Reis-Jr et al. ^6^ evaluated in a randomized study, by in vitro and in vivo microbiological analysis, the effects of photodynamic antimicrobial therapy (PAmT) on tibial surgical bone defects in rats infected by *Staphylococcus aureus* using bacterial counts carried out immediately and after 30 days after treatment as the outcome measure. The in vivo study PAmT group presented a bacterial reduction of 97.4%. Furthermore, the PAmT using toluidine blue effectively reduced the number of *S. aureus* in both in vitro and in vivo studies. Although PDT seems to be promising as adjuvant therapy in reducing bacteria in root canal treatments, reviews of the literature ^10^ and systematic reviews ^11^ found that no reliable protocol has been developed for light parameters, photosensitizers, and exposure time. Also, comparing results in a meta-analysis is still not possible because of the lack of standardized study methods.

Besides, the antimicrobial strategies used to sanitize the root canal must preserve the integrity of the dentin structure. The effect of the heat generated by lasers has been the subject of several studies ^12^ ^,^ ^13^. Formations similar to lavas ^14^, carbonization and dentin fusion ^15^, melting and recrystallization / resolidification of dentin, and changes in the morphology of the dentinal tubules ^16^ have been discussed after using the laser. The scanning electron microscopy (SEM) is a tool that enables analysis at high magnification and resolution, capable of detecting minor morphological changes in root dentin ^7^.

The parameters for effective laser action remain to be established. PDT success seems to be associated with several factors, such as bacterial sensitivity, type of photosensitizer, time for photosensitizers to penetrate bacteria, laser power, and duration of light application. This study evaluated the effect of PDT on the *Enterococcus faecalis* biofilm of infected root canals through microbiological analysis and scanning electron microscopy.

## Materials and Methods

### Bacterial strain

The bacterial strain used was *E. faecalis* (ATCC 29212) inoculated in 7mL of brain-heart infusion (BHI, Difco Laboratories, Detroit, MI) and incubated at 37^o^C for 24 hs. After that, 1(L of the solution was seeded on the surface of BHI agar incubated under the same conditions. Bacterial cells were resuspended in saline solution to a final concentration of about 3X10^8^ cells.mL^-1^, adjusted to a 1.0 McFarland standard, whose standard value using a UV spectrophotometer (Spectrophotometer Model Nova 1600 UV, Piracicaba, SP, Brazil) was 0.137nm.

### Sample preparation

Twenty-one extracted single-rooted human teeth with intact cement were provided by patients 18 years or older, extracted for periodontal or prosthetic reasons in the School of Dentistry of the Federal University of Goiás. Teeth with root canal treatment, obliterated canals, or root dilacerations were excluded. The methodology used was modified from a previous study ^17^.

Extracted teeth were kept in a 0.2% thymol solution. Before preparation, teeth were immersed in 5% NaOCl (Fitofarma, Lt. 20442, Goiânia, Brazil) for 30 min to remove organic tissues. Buccolingual and mesiodistal periapical radiographs were obtained using radiographic film (Eastman Kodak Comp., USA) to confirm the presence of a single root canal and the absence of anatomic variations, obliterations, or root canal treatment.

The crowns were removed under continuous air/water spray using an Endo-Z bur (Maillefer, Ballaigues, Switzerland) and a high-speed handpiece at an angle of 90 degrees to the long axis of the tooth. Root length was standardized at 16mm.

 Root canal patency was achieved using a K-Flex file #15 (Maillefer, Ballaigues, Switzerland) and confirmed by the direct visualization of the instrument's tip at the apical foramen. The apical diameter of root canals in all specimens was prepared using a BR5 instrument #40.04 (BioRace, FKG Dentaire, Swiss Dental Products, La Chaux-de-Fonds, Switzerland), and 3 mL of 2.5% NaOCl was used for irrigation at each instrument change.

 Paper points #40 were used to dry the canals, which were then filled with 17% EDTA (pH 7.2; Fórmula e Ação, São Paulo, Brazil) for 3 min to remove the smear layer. After that, the specimens were autoclaved for 3 min at 120^o^C and randomly divided into 5 experimental groups and 2 control groups (Table 1). The Research Ethics Committee of Federal University of Goiás approved this study (Protocol #19811113.0.0000.5083).

**Table 1:** Protocols of the groups of the study.

Groups	Protocols
1	S1+ RCP using NiTi rotary instruments and 2.5% NaOCl + 17% EDTA (3 min) + S2 + PDT (3 min) with 0.01% MB using a pre-irradiation time of 5 min + S3
2	S1+ RCP with stainless steel hand files and 2.5% NaOCl + 17% EDTA (3 min) + S2 + PDT (3 min) with 0.01% MB using a pre-irradiation time of 5 min +S3
3	S1+ RCP using NiTi rotary instruments and 2.5% NaOCl + 17% EDTA (3 min) + S2
4	S1 + 2.5% NaOCl irrigation + 17% EDTA (3 min) + S2
5	S1 + PDT (3 min) with 0.01 % MB using a pre-irradiation time of 5 min + S2
6	S1 - Negative control
7	S1 - Positive control

S1- sample collected before RCP (root canal preparation); S2 - sample collected after root canal sanitization - RCP or root canal irrigation (G4) or PDT (G5); S3 - sample collected after all procedures. MB - methylene blue solution.

### Preparation of experimental platforms and biofilm

For the experimental platform, the cervical portion of each specimen was connected to a 1.5mL polypropylene Eppendorf tube (Cral, São Paulo, Brazil) that had the bottom removed for this adaptation. A cyanoacrylate adhesive (Super Bonder, Itapevi, Brazil) was used to seal the connection, further fully covered with two layers of nail polish (Max Factor, Cosmetics and Fragrances, London, UK) to prevent infiltrations.

The specimens connected to the Eppendorf tubes were sterilized using 5% NaOCl for 30 min. After that, each set was attached to a 20mL flask with a perforated lid containing 10mL BHI culture medium (BHI; Difco Laboratories, Detroit, USA). The apical portion of the specimen was immersed during all contamination time. To ensure sterilization, the sets were incubated at 37^o^C for 48hs. After that, no bacterial growth was observed.

The biological marker described above was used to form the biofilm. The bacterial strain was inoculated in 7mL of BHI and incubated at 37^o^C for 24hs. Twenty-four hours before specimen inoculation, bacteria were again cultured on the surface of BHI agar and incubated as described. Bacterial inoculate was obtained by resuspending cells in saline solution to a final concentration of about 3X10^8^ cells mL^-1^, adjusted to #1 McFarland turbidity standard for spectrophotometry.

For sample contamination, 5mL of sterile BHI was mixed with 5mL of the bacterial suspension, and the samples in the experimental groups (n=18) were inoculated with *E. faecalis* using sterile syringes whose volume was enough to fill the root canal. The procedure was repeated for 60 days, every 72 hs using pure culture, and adjusted to the #1 McFarland standard. Specimens were kept at 37^o^C in a microbiological incubator.

After biofilm formation, root canals were dried and filled with sterile distilled water. Paper points sterile #40 (Tanari, Tanariman Indústria Ltda., Manacaru, Brazil) were placed in the canals and kept for 3 min for the first microbiological sample collection (S1). Each sample was collected using three paper points later immersed in 7mL of Letheen broth and two neutralizers, Tween 80 and sodium thiosulfate (P.A., Laboratório Art, Campinas, Brazil) at recommended concentrations, followed by incubation at 37˚C for 48hs. Before root canal preparation (RCP), the apical foramen of each specimen was sealed with acrylic resin.

 In Group 1, root canals were prepared with BioRace rotary files #50.04 and #60.02 (FKG Dentaire) which were discarded after three uses. During preparation, 3mL of 2.5% NaOCl was used for irrigation at each instrument change and the end of the RCP. Paper points sterile #60 were used to dry the canals, which were then filled with 17% EDTA (pH 7.2; Fórmula e Ação, São Paulo, Brazil) for 3 min to remove the smear layer. After that, material for S2 was collected as described for S1.

PDT consisted of irrigation of root canals with 1mL 0.01% methylene blue solution (Instituto Clemente Estable, Montevideo, Uruguay), using a pre-irradiation time of 5 min, followed by laser diode (InGaAlP red spectrum low power) application using a 600-(m optical fiber (660 nm+ 10nm, 100 mW OF OUTPUT POWER, Class III B, DMC Therapy XT, São Carlos, Brazil) for 3 min (deposited energy = 36 J). The optical fiber was placed 5 mm short of the apex and moved in spirals to about 3mm in the cervicoapical and apicocervical directions during laser application. During all the study, applications were made by the same operator wearing laser safety eyewear for protection against the effects of the laser on the eyes. The third sample (S3) was then collected as described above.

 In Group 2, root canals were prepared with stainless steel K-files #45 to #60 (Maillefer, Ballaigues, Switzerland). During RCP, 3mL of 2.5% NaOCl was used for irrigation at each instrument change and the end of the RCP. Paper points sterile #60 were used to dry the canals, which were then filled with 17% EDTA (pH 7.2; Fórmula e Ação, São Paulo, Brazil) for 3 min to remove the smear layer. After that, material for S2 was collected as reported above. The same PDT protocol as Group 1 was used, and S3 was then collected.

In Group 3, root canals were prepared as described for Group 1, and immediately after that, root canals were dried with paper points sterile #60 and filled with 17% EDTA (pH 7.2; Fórmula e Ação, São Paulo, Brazil) for 3 min to remove the smear layer. After that, material for S2 was collected as reported above.

 In Group 4, root canals were irrigated with 30mL of NaOCl, dried with paper points sterile #60, and filled with 3mL of 17% EDTA for 3 min. After that, material for S2 was collected as reported above. In Group 5, material for S1 was collected, and the same PDT protocol as Group 1 was applied immediately, without root canal preparation, followed by microbiological collection (S2).

In Group 6, material for S1 was collected to confirm specimen sterilization, and in Group 7, material for S1 was collected to verify specimen contamination. All roots were stored in a 2.5% buffered glutaraldehyde solution (pH 7.2) for 7 days for SEM preparation and analysis.

### Microbiological analysis

After the microbiological samples were collected, the tubes with paper points immersed in culture medium were transferred aseptically to a microbiological incubator at 37^o^C and kept there for 48 hrs. Then, all media were subcultured in other tubes with 7 mL BHI (Difco Laboratories, Detroit, MI), and culture media were stored as described above.

Bacterial growth was analyzed according to culture medium turbidity, and the presence of bacteria was evaluated using UV spectrophotometry (Spectrophotometer Model Nova 1600 UV, Piracicaba, Brazil), whose standard value was 0.137 nm to a final concentration of about 3X10^8^ cell.mL^-1^.

### Preparation for SEM analysis

After fixation in buffered glutaraldehyde, specimens were sectioned to expose the root canal. Two longitudinal grooves were made on each side along the root length using a diamond-coated disk (KG Sorensen Ind. Com., São Paulo, Brazil) at low rotation and air/water spray, making sure that the internal part of the canal was not touched. Next, a chisel was used to split the samples carefully along the buccolingual axis. Samples were dehydrated in 70%, 95%, and 100% alcohol solutions. The roots were kept for 30 min in each solution, which was refreshed every 10 min. CO_2_ critical point drying (Autosamdri®, 815, Series A) was performed before sputter-coating with gold (Denton Vacuum, Desk V).

The images were obtained using SEM images (Jeol, JSM 6610, equipped with EDS, Thermo Scientific NSS Spectral Imaging, Tokyo, Japan). The surface of the root canal was examined throughout the length of the two samples of each root, beginning in the cervical third and ending in the apical. The qualitative evaluations were carried out at magnifications 1,600X and 5,000X to describe the effect of PDT on the dentinal structure of the root canal.

### Statistical analysis

Nonparametric statistical techniques were applied using the SPSS 18.0 software (SPSS Inc. Chicago, IL) and Microsoft Excel^®^ 2010 spreadsheets. Variables were described as median, minimum and maximum. The Wilcoxon test was used to compare samples collected from the same specimens. The Mann-Whitney test was used for the comparisons between groups. The level of significance was set at 5%.

## Results

All experimental groups' microbiological samples (S1, S2, and S3) presented *E. faecalis*. Table 2 shows the median values of optical density (nm) in the culture medium for each group.

In Groups 1 and 2, the median of the optical density of culture media was lower in S2 than in S1, and the percentage variation of the median was 28.7% and 93.7%, respectively. Still, there were no statistically significant differences (p> 0.05). After PDT in groups 1 and 2, there was an additional reduction of 90.0% and 92.0%, respectively, in the optical density of the culture medium in S3 (p>0.05).

**Table 2:** The median value of optical density (nm) and percentage variation (%) of the microbiological samples (S1, S2, and S3).

Groups	Microbiological Samples							
	S1	OD (nm) Median	S2	OD (nm) Median	Variation %	S3	OD (nm) Median	Variation %
I	+++	0.314	+++	0.224	Reduction 28.70%	+++	0.026	Reduction 90.0%
II	+++	0.394	+++	0.025	Reduction 93.70%	+++	0.004	Reduction 92.0%
III	+++	0.224	+++	0.086	Reduction 61.61.%	NA	NA	NA
IV	+++	0.244	+++	0.093	Reduction 61.89 %	NA	NA	NA
V	+++	0.317	+++	0.340	Increase 3.2%	NA	NA	NA
VI	---	0.00	NA	NA	NA	NA	NA	NA
VII	+++	0.315	NA	NA	NA	NA	NA	NA

*+++ presence of bacteria; --- absence of bacteria; NA - not applicable; Mann-Whitney test; OD - Optical Density.*

In Group 3 e 4, the analysis of culture media at S2 revealed a reduction of 61.61% and 61.89%, respectively, in optical density compared to S1(p>0.05). In Group 5, the analysis of culture media at S2 revealed an increase of 3,2% in optical density compared to S1, but the differences were not statistically significant (p=0.593). In negative and positive controls, the optical density of culture media was 0.00nm and 0.315nm, respectively.

**Figure 1:**
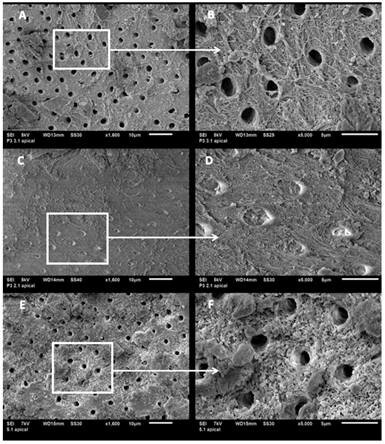
SEM images (1,600x and 5,000x) of the PDT groups. G1 shows irregularities in the contour and exposure of collagen fibers (A and B); G2 presents evident obliteration of dentinal tubules, as the melting point and recrystallization (C and D); G5 shows the irregular surface with projections of peritubular dentin (E and F).

 The effect of PDT on the dentinal structure of the root canal in Groups 1, 2, and 5 observed in SEM images is presented in Figure 1. In the samples of G1, dentin showed regular surface in the cervical and apical thirds and irregularities in the middle third. In all thirds, openings were observed from reduced dentinal tubules with the most evident obliteration in the cervical and apical thirds. The presence of the smear layer and debris was verified. The melting point and recrystallization were more pronounced in the apical third. In the samples of G2, the dentinal tubules showed up obliterated with a noticeable change in the contour of their openings and projection in relief evident peritubular dentin in the middle third. In addition, the melting point and recrystallization were observed. In the samples of G5, the irregular surface was seen in the cervical third, melting point, and recrystallization with projections of peritubular dentin, changing the shape of the openings of the dentinal tubules. In the middle and apical thirds, dentin structure was not visible.

 The dentinal tubules opening was preserved in all thirds analyzed of the samples of G3, which showed a regular dentin surface. Therefore, it was verified a presence of debris. The samples of G4 exhibited dentin surface with irregular aspect in the cervical third, with the erosion of inter- and peri-tubular dentin with union dentinal tubules openings. Dentin's structure was not visible in the middle and apical thirds. Debris was evident in all thirds.

In the samples of G6, dentinal tubules presented open and regular contour, with slight exposure of collagen fibers inside. The intertubular dentin proved to be regular, free of smear layer, bacteria, and debris. In the samples of G7, a dense biofilm was found covering the dentin surface throughout the length of the root canal. The openings of dentinal tubules did not show up visible.

## Discussion

The antibacterial strategies tested on infected root canals showed reductions, as PDT with 0.01% methylene blue PS using a pre-irradiation time of 5min, but *E. faecalis* was not completely eradicated. Souza et al. ^4^ found an additional bacterial reduction of 12.28% after instrumentation and irrigation with 2.5% NaOCl using PDT on canals infected with *E. faecalis*. These variations in results may be explained by the PDT parameters adopted once the irradiation time by the laser used was 3min. Silva et al. ^18^ found better results with laser application for 1 and 2 min on *E. faecalis* suspension, while Yildirim et al. ^19^ did not find any differences in the reduction of the number of bacteria in a study that also used root canals prepared with rotary instruments and infected with *E. faecalis*, after applied PDT using laser irradiation for up to 4 min.

Cationic phenothiazine dyes are the most frequently used PS due to their phototoxic efficiency against a wide spectrum of microorganisms, and also, they need less energy to achieve the same result as other PS ^9^. Moreover, methylene blue has satisfactory results and a remarkable capacity to penetrate the polymeric matrix of bacterial biofilm because of its hydrophilic nature, low molecular weight, cationic nature, and water solubility, which promotes better adhesion to anionic structures, such as teichoic acid, in the most internal portions of biofilm ^20^. PS concentration may also affect PDT results. An in vitro study ^3^ found that 0.01% methylene blue generated a more considerable amount of singlet oxygen (^1^O_2_) and, therefore, had a better bactericidal effect against *E. faecalis*. In this study, 0.01% methylene blue was applied for 5min.

The physical properties of antimicrobial solutions are directly associated with their capacity to penetrate areas that are difficult to access in the root canal so that they can be absorbed by bacterial cells. George and Kishen ^21^ described the photophysical, photochemical, and photobiological characteristics of different methylene blue formulations for PDT. Although bacteria better absorbed it when dissolved in water, the higher penetration in dentinal tubules and better effectiveness against *E. faecalis* 4-day biofilm in root canals was better when a combination of glycerol, ethanol, and water was used. The polysaccharides in biofilm, which adhere to the substrate and the cells, may act as a barrier to the penetration of PS into bacteria, preventing the formation of reactive oxygen products, which are responsible for bacterial death. The trapped PS absorbs photons and generates harmless reactive oxygen products, reducing the number of photons absorbed by PS located inside bacterial cells ^22^. A more considerable amount of singlet oxygen directly affects extracellular molecules due to its high chemical reactivity, which makes polysaccharides in the extracellular polymeric matrix of bacterial biofilm susceptible to PDT action ^23^. In the presented study, an aqueous methylene blue solution was used, which might have limited its bactericidal effect because of the high surface tension of water and prevented the contact of PS with the bacteria adhered to areas difficult to access and to deeper portions of dentinal tubules.

This study evaluated the bactericidal effect of PDT without previous root canal preparation. In group 5, the optical density of the culture medium increased after PDT. Bago et al. ^24^ evaluated the effect of PDT on root canals infected with *E. faecalis* and found that, according to CFU counts, bacterial reduction reached 99.99% even when no previous preparation was used. Similar results were reported, with bacterial decreases of 96.6% ^25^ and 77.5% ^26^. These results might be explained by the different laser powers used, 0.2W and 1W, or by the fact that PDT was applied to immature biofilm. PDT is a complementary protocol in the RCP and not an alternative to it. Only instrumentation and irrigation can rupture biofilm structures, making bacteria more susceptible to PDT action ^5^.

In the present study, the analysis of the effect of PDT on the dentinal structure using SEM showed aspects of melting and recrystallization of dentin in all images of the samples in which the laser was used. In the infrared spectrum, the energy is absorbed by the mineral structures of the dentine, such as the phosphate and carbonate, disorganizing the crystalline arrangement due to the thermal ablation, thus promoting the melting in the dentin tissue ^27^. The recrystallization process occurs after the rapid cooling of the dentin, responsible for some changes in the structure of hydroxyapatite, such as the formation of tricalcium phosphate ^28^.

Soukos et al. ^29^ observed the increase in temperature, which showed elevation above 45(C when 1W power was applied, but it did not exceed body temperature at the external root surface. These changes have been reported in studies with high power laser, which has been responsible for thermal damages such as carbonization, dentin melting, and subsequent recrystallization ^30^. In the present study, PDT was used with a low-power source (100mW) and lower heat generation, but the dentin structure observed the same changes.

Areas of intertubular dentin erosion and union of dentinal tubule entries were observed in the present study in G4 specimens and may be attributed to the use of 2.5% NaOCl and EDTA, with similar changes found in another study ^31^.

SEM images showed dentinal tubules with altered contours in the samples of G1 and 2, and with obliterated entries. The smear layer that remains on the dentin after irrigation with NaOCl presents high mineral content, and under the effect of the heat generated by the laser, can be fused obliterating the entrance of the dentinal tubules ^32^. These ultrastructural changes of the dentin, reported in the literature after the use of PDT, have been directly related to the increase in temperature, which depends on the power, frequency, and form of laser application ^33^.

Despite simulating a clinical condition with a mature biofilm of a microorganism of great relevance to endodontic failure, it is an in vitro study. Thus, the extrapolation of the results must be carried out cautiously. However, the study represents an advance in understanding processes related to root canal sanitation and relates PDT to yet another supporting factor in this process of significant clinical relevance.

Although PDT has promising results as a contribution to the bacterial reduction in root canals, it still has limitations, such as its difficulty of penetration into biofilm ^25^, as well as the effects of tissue inhibitors and efflux pump inhibitors ^9^. In addition, the complex anatomy of root canals and dentin porosity may affect results ^20^. The variety of parameters associated with a light source and PS used in PDT are essential factors and should be investigated in further studies, once these factors seem to affect treatment efficiency.

## Conclusion

PDT after root canal preparation using the rotary system or manually, associated with 2.5% NaOCl, was not able to completely eliminate *E. faecalis* mature biofilm present in the root canal.
